# Hypersensitivity Reaction Post Laparoscopic Cholecystectomy Due to Retained Titanium Clips

**DOI:** 10.7759/cureus.26167

**Published:** 2022-06-21

**Authors:** Fabrice Yabit, Lauren Hughes, Bertha Sylvester, Frederick Tiesenga

**Affiliations:** 1 Surgery, Saint James School of Medicine, Chicago, USA; 2 General Surgery, West Suburban Medical Center, Chicago, USA

**Keywords:** titanium, retained clips, hypersensitivity reaction, general surgery, laparoscopic cholecystectomy

## Abstract

Titanium is an inert metal that has many medical uses and applications because of its biocompatibility. However, titanium is not completely devoid of adverse reactions. This was the case of a 55-year-old female who presented following a laparoscopic cholecystectomy with right upper quadrant pain, diarrhea, nausea and eventual neuralgia in the right lower extremity. This led to a series of diagnostic procedures including an esophagogastroduodenoscopy (EGD), a colonoscopy, a CT scan, and an abdominal ultrasound, all of which proved to be futile. The patient was eventually formally diagnosed with hypersensitivity reaction due to titanium clips and underwent a diagnostic laparoscopy, adhesiolysis, and removal of foreign body under fluoroscopy. A total of four clips were identified and removed during the procedure. Repeat x-ray showed no evidence of further clips in the right upper quadrant nor throughout the abdomen. The patient was discharged the same day and showed improvement of symptoms one week later during the post-operative follow-up visit.

## Introduction

Titanium clips are commonly used in surgical procedures. This metal has significant advantages as it is neither toxic to body tissues nor causes hypersensitivity reactions [1]. Despite its biocompatibility, there have been various cases and patients who have had residual side effects after surgical clips were placed. Although rare, common reactions include but are not limited to, erythema, necrosis, eczema and urticaria [2]. Wegner et al. reported a case of a 65-year-old female who presented with breast pain after titanium clip placement during a breast biopsy. The symptoms eventually resolved after vacuum-assisted removal of the clips and further histology revealed lymphocytic infiltration of the tissue [3]. Another case by Hosoki et al. describes a 69-year-old male who developed eczema after placement of titanium screws for a leg fracture. Full remission was observed once the titanium implants were removed [4]. In this case, we present a patient with multiple adverse effects observed following a laparoscopic cholecystectomy case where titanium surgical clips were placed.

## Case presentation

This case involves a 55-year-old female with a past medical history significant for colonic polyps, gallbladder disease, gastritis, gastroesophageal reflux disease and a thyroid nodule who presented with chronic upper right quadrant pain and other nonspecific symptoms following a laparoscopic cholecystectomy in 2017. Her medications included alprazolam, cetirizine, omeprazole, vitamin C, vitamin D, dicyclomine, and triamcinolone. The patient's known allergies included amitriptyline, sulfonamides, hydrocodone, latex, nickel and contrast media. The postoperative course was marked by worsening right upper quadrant pain, abdominal pain, diarrhea, nausea, general fatigue and body aches which eventually progressed to neuropathic pain along the right leg.

The patient sought out medical advice shortly after the symptoms began and was referred to a variety of specialists by her primary care physician. Numerous diagnostic tests were performed, all of which were inconclusive. This included two colonoscopies in 2017 and 2020 and three esophagogastroduodenoscopies (EGD) in 2017, 2018, and 2020. The patient continued live with the pain, often medicating with antihistamines for symptomatic relief until she found an online support group for patients who had chronic conditions such as hers following a surgical intervention for gallbladder disease.

**Figure 1:**
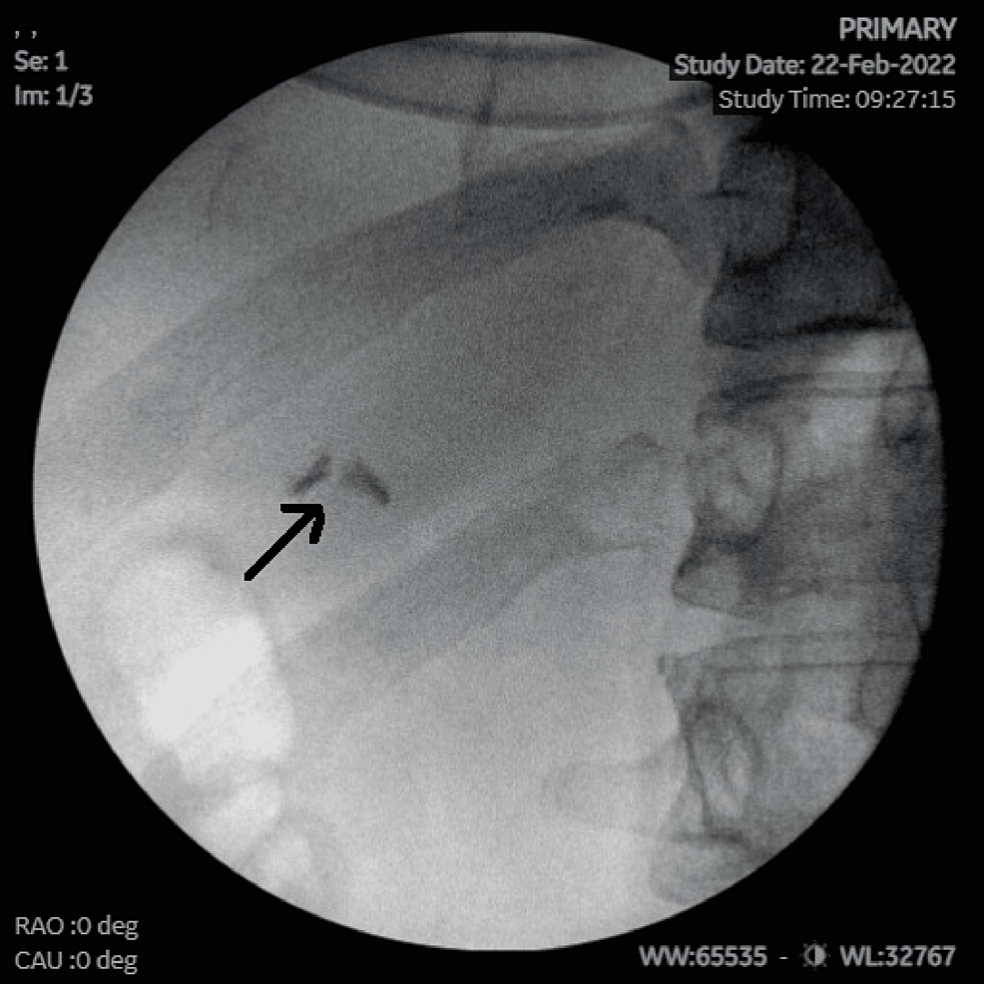
Intraoperative fluoroscopy showing surgical clips in the right upper quadrant of the abdomen.

**Figure 2:**
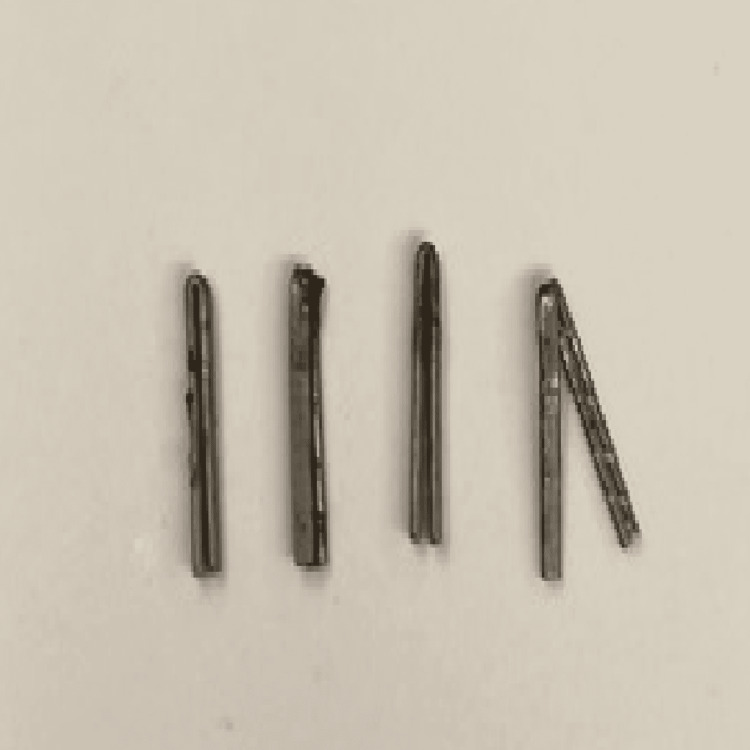
The extracted foreign bodies included four titanium staples with no apparent abnormalities

**Figure 3:**
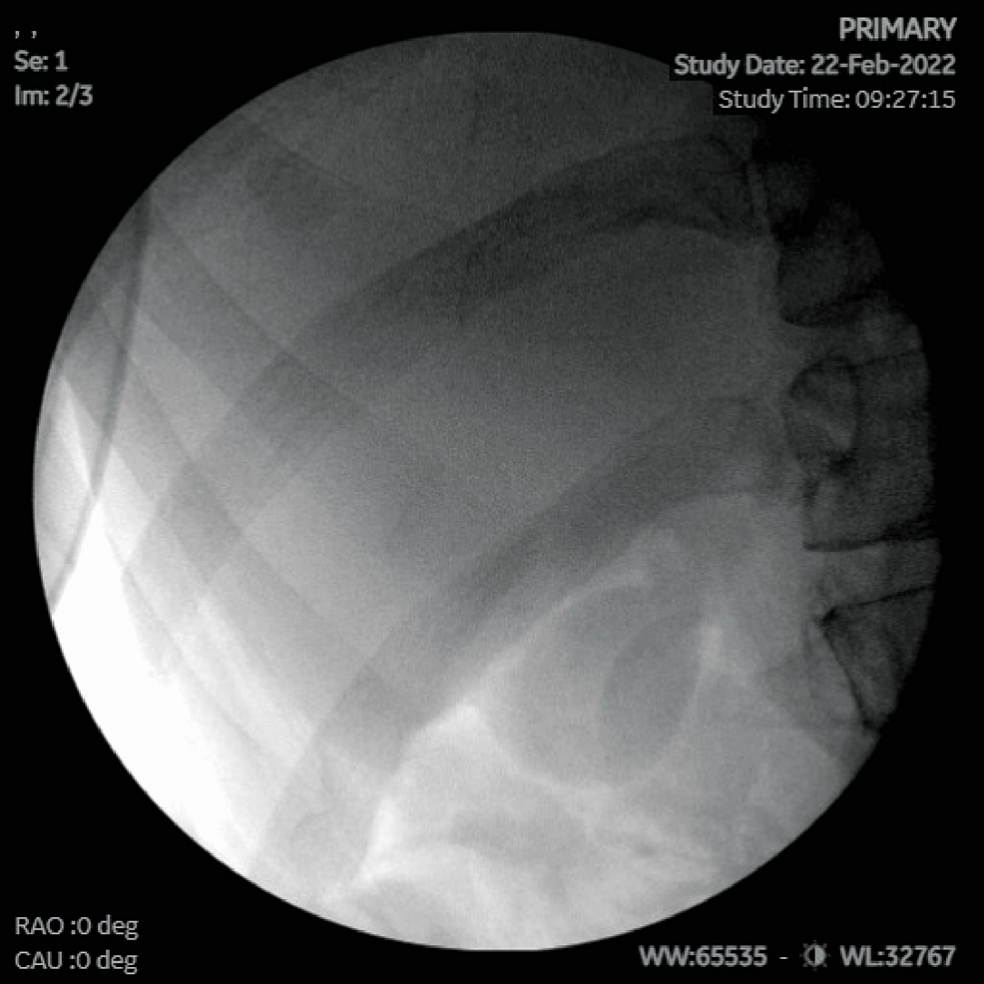
Repeat fluoroscopy confirming the complete removal of the surgical clips

She was referred to a general surgeon who had previously treated such patients, many of them to complete resolution of symptoms. Following a surgical consultation, a formal diagnosis of hypersensitivity reaction to titanium clips was given. The patient eventually underwent a diagnostic laparoscopy, adhesiolysis, and removal of the foreign bodies under fluoroscopy. Adhesions to the gallbladder fossa were removed with cautery, fluoroscopy was performed showing clips in the right upper quadrant (Figure 1). The area of the right upper quadrant was dissected, the clips were identified, all were removed and accounted for (Figure 2). Repeat x-ray showed no evidence of clips in the right upper quadrant or throughout the abdomen (Figure 3).

The patient was successfully discharged home to family care the same day without any postoperative complications. A phone interview on postoperative day one and an outpatient follow-up visit on the seventh postoperative day were conducted, during which the patient expressed minimal abdominal pain and gradual relief of presenting symptoms. 

## Discussion

Titanium hypersensitivity is a rare occurrence that can be seen in patients who receive various types of implants. It is suggested that these cases are due to a type IV hypersensitivity reaction, a common allergy mediated by CD4+ helper T-cell [5].

According to Baumann and Crist, clusters of differentiated 4 (CD4) helper T-cells become activated, release inflammatory cytokines and activate macrophages that kill targeted cells [6]. The patient reported nonspecific symptoms including worsening right upper quadrant pain, chronic abdominal pain, diarrhea, nausea, generalized fatigue, lethargy, anxiety, body aches and neuropathic pain. Other nonspecific symptoms commonly reported in postoperative hypersensitivity reactions include myalgia, joint pain and tenderness, mental fogginess, irritable bowel syndrome, vision changes, hypertension and hand tremors [7]. 

It is of note that these symptoms are different from those typically seen in hypersensitivity reactions such as erythema, itchiness, blistering, and exudate. With the growing prevalence and knowledge regarding such cases, physicians should be aware of the nonspecific symptoms of hypersensitivity reactions when evaluating patients for abdominal pain and other potential atypical symptoms in surgical patients with application of titanium clips.

When examined with other similar cases regarding postoperative metal hypersensitivity reactions, this case reflects the usual diagnostic delay and the unnecessary procedures causing additional physical and emotional stress for patients. Throughout the postoperative course, the patient was given a diagnosis of gastritis and gastroesophageal reflux disease (GERD). Medications prescribed to her during this timeframe included cetirizine, alum hydroxide-mag carbonate and omeprazole magnesium. 

The patient reported improvement of symptoms on the first postoperative day following the removal of the four titanium clips. The patient continued to report substantial improvement in symptoms on the seventh postoperative day. A similar case by Shah et al. presented a 54-year- old female with a past surgical history of cholecystectomy in 2007 who presented with multiple nonspecific symptoms. Subsequent allergy testing showed positive hypersensitivity to cobalt, nickel, and mercury, and all symptoms resolved six weeks after removal of her metallic clips. The patient reported by Shah et al. was given numerous diagnoses early in the course of treatment including early menopausal symptoms, hypothyroidism and hypochondria [7].

Titanium implants that present with nonspecific misdiagnosed symptoms may put both the patients and the healthcare system to additional expenses, but more importantly cause grave distress to these patients. Therefore, with patients who have had prior surgical intervention due to gallbladder disease, there should be an emphasis on placing hypersensitivity reactions higher on the list of differential diagnoses.

Although contact nickel allergy is preventable, according to Thyssen et al., 17% of women and 3% of men in the general population display this allergy. Our 55-year-old patient has a history of both nickel and contrast allergy [8]. According to Neves et al., titanium implant-related complaints could be due to localized irritant-mediated inflammation arising from leachable agents rather than a titanium metal allergy [9]. Our patient did not receive patch or other allergy testing for titanium pre cholecystectomy, though she has a known history of metal (nickel) allergy, as well as multiple other allergies, including IV contrast. Based on this report, we recommend preoperative allergy testing for patients with history of known allergies, especially those with metal allergies to prevent further complication or aid in diagnosis and treatment [10].

## Conclusions

Titanium has been used for years in dental implants and has shown high level of success because of its biocompatible resistance to corrosion and mechanical properties. It is widely successful because it is thought to be neither nontoxic nor cause hypersensitivity reactions. However, in a subset of the population, titanium clips used in surgical procedures have led to further complications requiring surgical removal. Adequate history-taking with a focus on any allergy history should alert the practitioner to perform patch and allergic testing before placement of titanium clips.
